# A technique to maintain pneumoperitoneum and allow easy inspection of the abdomen after specimen delivery in laparoscopic colorectal surgery

**DOI:** 10.1308/003588412X13373405385214e

**Published:** 2012-07

**Authors:** H Travers, S Mansfield

**Affiliations:** Royal Devon and Exeter NHS Foundation Trust,UK

## BACKGROUND

The majority of laparoscopic colorectal procedures require utility incisions to be made for specimen delivery. Resealing this incision to allow further inspection of the abdominal cavity can be problematic. This simple technique allows a good seal and facilitates insertion of single or multiple ports.

## TECHNIQUE

**Figure 1:**
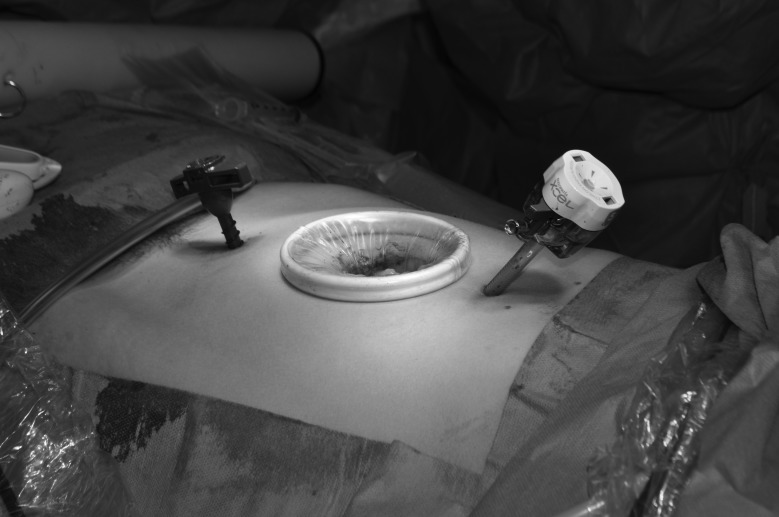
Alexis® retractor in extended port site incision

**Figure 2:**
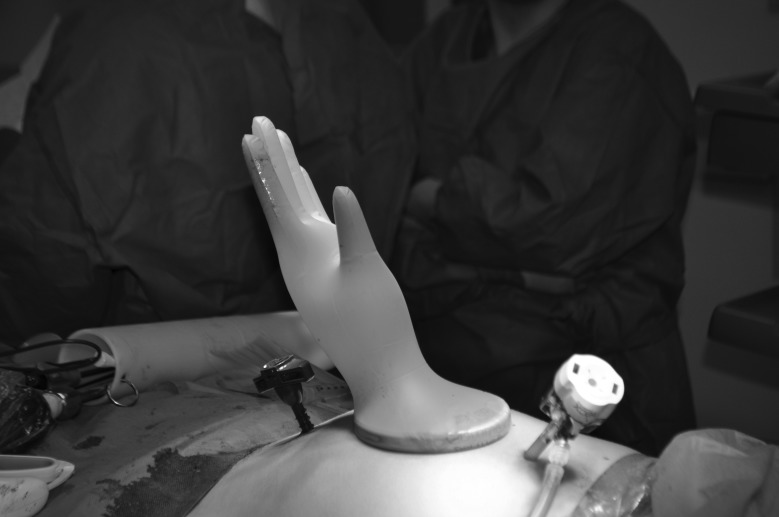
Glove stretched over the retractor, providing a seal

**Figure 3:**
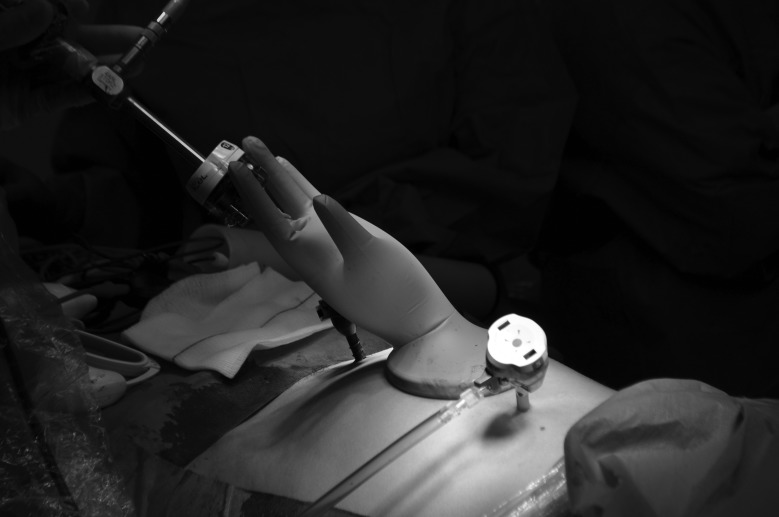
Glove port with camera inserted

A small Alexis® retractor (Applied Medical, Rancho Santa Margarita, CA, US) is placed in the extended port site incision prior to specimen removal (Fig 1). This technique can also be used at a stoma site. Once the specimen is removed, stretch a sterile size 6½ glove over the retractor and re-establish pneumoperitoneum. A perfect seal will be obtained (Fig 2). If the port is still required, cut the tip off any finger of the glove and reinsert the trocar, placing a tie on the glove to maintain the seal around the trocar. The trocar can be used as normal (Fig 3).

## DISCUSSION

This technique is the best we have used for reinsufflation and reinsertion of ports. It allows a port site incision to be extended for specimen delivery and prevents the need for further incisions/port sites as often required. The seal obtained establishes pneumoperitoneum easily, allowing adequate visualisation of the peritoneal cavity either for inspection or further procedures such as forming anastomoses. It can also be used as a primary port site, particularly in procedures where a stoma has had to be mobilised as the first part of a procedure. The use of this technique minimises the number of incisions required while maintaining adequate and safe visualisation and access.

